# Competitive ubiquitination activates the tumor suppressor p53

**DOI:** 10.1038/s41418-019-0463-x

**Published:** 2019-12-02

**Authors:** Xingyao Li, Mengqi Guo, Lun Cai, Tingting Du, Ying Liu, Han-Fei Ding, Hongbo Wang, Junran Zhang, Xiaoguang Chen, Chunhong Yan

**Affiliations:** 1 Georgia Cancer Center, Augusta University, Augusta, GA 30912 USA; 2 College of Pharmacy, Yantai University, Yantai, Shandong China; 3 Department of Pharmacology, Institute of Materia Medica, Chinese Academy of Medical Sciences and Peking Union Medical College, Beijing, 100081 China; 4 Department of Pathology, Medical College of Georgia, Augusta University, Augusta, GA 30912 USA; 5 Department of Radiation Oncology, Ohio State University, Columbus, OH 43210 USA; 6 Department of Biochemistry and Molecular Biology, Medical College of Georgia, Augusta University, Augusta, GA 30912 USA

**Keywords:** Tumour-suppressor proteins, Molecular biology

## Abstract

Blocking p53 ubiquitination through disrupting its interaction with MDM2 or inhibiting the MDM2 catalytic activity is the central mechanism by which the tumor suppressor p53 is activated in response to genotoxic challenges. Although MDM2 is first characterized as the major E3 ubiquitin ligase for p53, it can also catalyze the conjugation of ubiquitin moieties to other proteins (e.g., activating transcription factor 3, or ATF3). Here we report that ATF3 can act as an ubiquitin “trap” and competes with p53 for MDM2-mediated ubiquitination. While ATF3-mediated p53 stabilization required ATF3 binding to the MDM2 RING domain, we demonstrated that ATF3 ubiquitination catalyzed by MDM2 was indispensable for p53 activation in response to DNA damage. Moreover, a cancer-derived ATF3 mutant (R88G) devoid of ubiquitination failed to prevent p53 from MDM2-mediated degradation and thus was unable to activate the tumor suppressor. Therefore, we have identified a previously-unknown mechanism that can activate p53 in the genotoxic response.

## Introduction

The tumor suppressor p53 is the guardian of the genome, and maintains genetic integrity by regulating expression of an array of genes involved in a variety of cellular events (e.g., cell cycle progression, programmed cell death, and metabolism) [1, 2]. While the p53 tumor suppressor activity is tightly regulated at multiple levels, inhibition of MDM2-mediated ubiquitination that can drive p53 for proteasomal degradation serves as the central mechanism for p53 activation in response to genotoxic stress. Indeed, DNA damage-induced phosphorylation of p53 and MDM2 can dissociate p53 from MDM2 and stabilize the former by blocking its ubiquitination [3]. Oncogenic stress, on the other hand, stabilizes and activates p53 by inducing ARF, which in turn binds MDM2 and inhibits its catalytic activity [4]. Many other proteins that can bind either p53 or MDM2 utilize similar mechanisms to activate p53 upon genotoxic challenges [5]. The crucial role of MDM2 in the regulation of p53 activity is supported by the observations that knockout (KO) of the *Trp53* gene rescues mouse embryonic lethality caused by *Mdm2* loss [6, 7], and that overexpression of MDM2, or its homolog MDMX, frequently occurs in human cancers not harboring *TP53* mutations [8]. MDM2 is a RING-type E3 ubiquitin ligase, and its C-terminal RING domain functions to recruit charged E2 ubiquitin-conjugating enzymes (mainly UbcH5 family) [9] and prime the transfer of ubiquitin from E2s to substrates [10–12]. Although MDMX does not bind the E2-ubiquitin (Ub) complex, it forms a dimer with MDM2, stabilizes a closed, “folded-back” E2-Ub conformation, and thereby promotes Ub transfer [12]. Given the importance of p53 in tumor suppression, it is vital to fully understand the mechanism by which p53 is fine tuned in the genotoxic response.

Activating transcription factor (ATF3), like p53, is a common stress sensor [13]. Consistent with its responsiveness to a wide range of cellular stress, ATF3 is involved in many physiological and pathological events (e.g., myocardial repair, viral infections, diabetes, and immune response). Although ATF3 may regulate cancer progression and metastasis in a context-dependent manner [14, 15], we have shown that *Atf3*^*−/−*^ mice are prone to spontaneous tumorigenesis and *Atf3*-deficient cells are genetically unstable [16]. While ATF3 can promote ATM activation and regulate the DNA damage response by stabilizing the histone acetyltransferase Tip60 [17], the ATF3 tumor suppressor activity is also attributable to its ability to directly activate p53 upon DNA damage [18]. Indeed, ATF3 not only stabilizes p53 [18], but colocalizes with p53 at a number of genomic sites for transcriptional regulation [19]. Mechanistically, ATF3 directly binds p53 at its C-terminus, and blocks its ubiquitination mediated by MDM2 [18]. While genetic evidence has confirmed that ATF3 can activate p53 upon γ-irradiation (IR) or oncogenic stress [16, 20], how ATF3 prevents p53 from MDM2-mediated ubiquitination remains unclear. The binding of ATF3 to the p53 C-terminus does not appear to shield the C-terminal residues from ubiquitination, nor disrupt the p53–MDM2 interaction [18]. Intriguingly, ATF3 also binds MDM2, and can be ubiquitinated by the latter protein [21]. Thus, ATF3 represents a group of proteins that can stabilize p53 but meanwhile are bona fide substrates for MDM2 [22]. ATF3 binds to the MDM2 RING domain responsible for the recruitment of E2-Ub [21]. This is distinct from p53, which binds to the MDM2 N-terminus distal to the RING domain and thus requires an additional domain (i.e., the MDM2 acidic domain) to promote its ubiquitination [23, 24]. As spatial proximity of E2s to substrates is important for ubiquitin transfer and ubiquitin chain elongation [25, 26], the binding of ATF3 directly to the MDM2 RING domain raises an intriguing possibility that ATF3 might compete with p53 for MDM2-mediated ubiquitination thereby activating the tumor suppressor. Here we present evidence supporting the notion that ATF3 can serve as an “ubiquitin trap” that activates p53 by competitive inhibition of p53 ubiquitination.

## Methods and materials

### Plasmid construction

Constructs expressing ATF3 C-terminal truncates were generated by amplifying desired fragments with PCR and subcloned into the HindIII/EcoRI sites of pcDNA3-FLAG. To generate chimeric JDP2(AB) and ATF3(JB) constructs, PCR was used to amplify ATF3 BR and JDP2 BR, and assembled with pcDNA3-FLAG by Gibson Assembly using a kit from New England Biolabs. Substitutions of single or multiple ATF3 residues were carried out by overlapping PCR using complementary primers spanning the mutated areas, and the PCR fragments were cloned into pcDNA3-FLAG. The K107R and K108R mutants were also PCR amplified and cloned into the HindIII/BamHI sites of the pTrcHis vector to express recombinant proteins for purifications. All of the mutated/chimeric constructs were sequenced to confirm that they contain desired sequences.

### Cell culture and transfections

H1299 and U2OS cells were obtained from ATCC, and cultured in RPMI 1640 and DMEM supplemented with 10% FBS, respectively. ATF3-knockout U2OS cells were generated by CRISPR/Cas9 as previously described [27]. To reconstitute with wild-type ATF3 and ATF3 mutants, ATF3-KO cells were transfected with FLAG-ATF3wt, FLAG-K107R, FLAG-K108R, or FLAG-R88G using Lipofectamine 2000 (Invitrogen) according to the manufacturer's instruction. Transfected cells were selected with 800 μg/ml of G418, and ATF3-expressing clones were identified by western blotting.

### Western blotting, co-immunoprecipitation assays, and GST-pulldown assays

These were carried out as described previously [18, 21]. Western blotting experiments were usually performed 2–3 times, and representative results are presented in figures. The following primary antibodies were used: ATF3 (sc-188X, 1:10,000), MDM2 (N-20, sc-813, 1:1000), p53 (DO-1, sc-126, 1:1000), and HA (sc-7392, 1:2500) from Santa Cruz Biotechnology; PUMA (2–16, 1:1000) from CALBIOCHEM; p21(556,431, 1:1000) from BD Pharmingen; β-actin (A2228, 1:10,000) and FLAG (F3165, 1:5000) from Sigma; and GFP (JL-8, 1:8000) from Clontech.

### In vitro and in vivo ubiquitination assays

In vitro ubiquitination assays were described previously [18, 21]. Briefly, ATF3 or p53 was in vitro translated using the TNT Quick Coupled Transcription/Translation System (Promega). 0.5 μl of in vitro-translated protein was incubated with 50 ng of E1, 210 ng of E2, 200 ng of GST-MDM2, 5 ug of ubiquitin, in the presence/absence of varying amounts of recombinant ATF3 in a buffer containing 40 mM Tri-HCl, pH 7.5, 5 mM MgCl2, 2 mM DTT, and 2 mM ATP (25 μl volume) at 37 °C for 90 min. The reactions were terminated by boiling in the SDS-loading buffer for 5 min before loaded for western blotting using the FLAG or p53 antibody. E1 (UBE1, #E-304), E2 (UbcH5a, #E2-616), and ubiquitin (U-100H) were purchased from Boston Biochem. For in vivo ubiquitination assays, H1299 cells transfected with FLAG-ATF3, MDM2, p53, and HA-ubiquitin were treated with 10 μM of MG132 overnight, and then lysed in the FLAG lysis buffer (50 mM Tris-HCl, pH 7.9, 137 mM NaCl, 10 mM NaF, 1 mM EDTA, 1% Triton X-100, 0.2% sarkosyl, and 10% glycerol). For ATF3 ubiquitination assays, cell lysates (1–2 mg) were incubated with 20 μl of anti-FLAG M2 affinity gel (Sigma) at 4 °C overnight. After extensive washes, agarose gels were loaded on spin columns (Affymetrix), and bound ATF3 was eluted with 20 μl of FLAG peptide at a final concentration of 100 μg/ml. ATF3 ubiquitination was determined by western blotting using the HA antibody. For p53 ubiquitination assays, cell lysates were incubated with 20 μl of the anti-HA affinity gel, and the HA-tagged proteins were then released by boiling the gel for 10 min in 30 μl of 2× SDS-loading buffer for western blotting using the p53 antibody.

### Flow cytometry and cell cycle analysis

Cells exposed to 10 Gy of IR were suspended in PBS, and fixed with cold 70% ethanol at 4 °C overnight. Cells were then washed with PBS, incubated in a solution containing 50 μg/ml of propidium iodide (Sigma) and 20 μg/ml of RNase A (Sigma) at 37 °C for 20 min, and analyzed on a BD LSR II cytometer. The data were analyzed by the FlowJo software, and *p* values were calculated by the Student’s *t* test.

## Results

### The ATF3 basic-region domain is required for increasing the p53 level

**Fig. 1:**
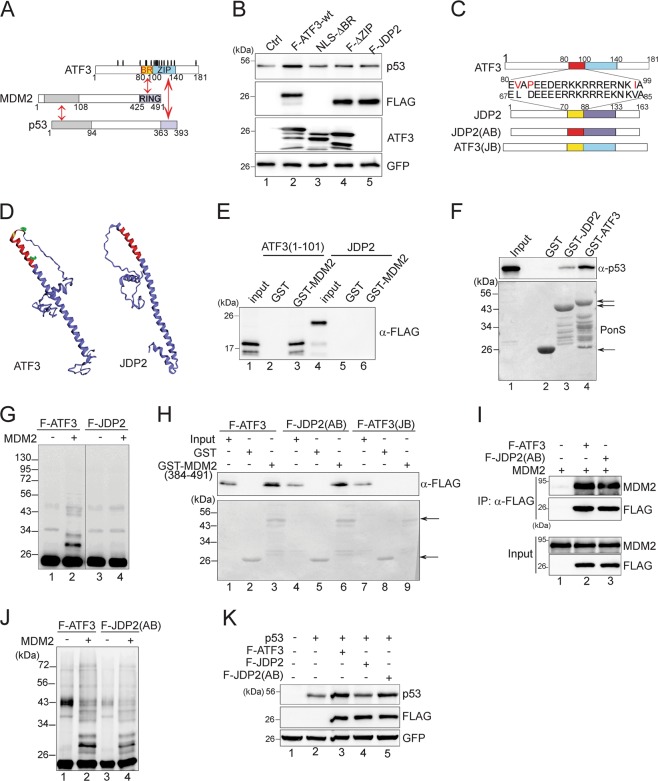
The ATF3 basic region is required for p53 stabilization. **a** Schematic representation of the ATF3 domains responsible for binding to MDM2 and p53. Red two-direction arrows indicate interactions. Positions of lysine residues are also marked as black ovals. **b** H1299 cells were transfected with p53, GFP, in the presence/absence of FLAG-tagged ATF3wt, JDP2, or ATF3 deletions as indicated for western blotting. The GFP level was determined for the control of transfection efficiency. **c** The sequence of ATF3 BR is compared with that of JDP2. **d** ATF3 and JDP2 structures predicted with the I-TASSER server. Note the similarity and the difference in the BR region (colored with red) between ATF3 and JDP2. The side chains of V81 and I98 are shown in green. P83 is colored in yellow. **e** FLAG-tagged ATF3(1–101) or JDP2 was in vitro translated, and incubated with immobilized GST-MDM2 (384–491) for GST-pulldown assays. The MDM2 C-terminal fusion was used in most of the experiments because it was better folded and expressed in *E. coli*. ATF3 efficiently binds to this C-terminal fragment [21]. **f** In vitro translated p53 was incubated with immobilized JDP2 or ATF3 for GST-pulldown assays. Arrows indicate GST or GST fusion proteins. **g** In vitro translated FLAG-ATF3 or FLAG-JDP2 was incubated with purified MDM2 and other ubiquitination reaction components for in vitro ubiquitination assays. Ubiquitinated proteins were detected with the FLAG antibody. **h** Indicated FLAG-tagged proteins were in vitro translated and incubated with GST-MDM2 (384–491) for GST-pulldown assays. **i** H1299 cells were transfected as indicated, and subjected to FLAG-IP for western blotting to detect MDM2 binding. **j** Indicated proteins were in vitro translated and subjected to in vitro ubiuquitination assays. **k** H1299 cells were transfected as indicated for western blotting

ATF3 binds to the C-termini of both p53 and MDM2 via its leucine-zipper domain (aa 102–139, ZIP) and basic-region domain (aa 80–100, BR), respectively [18, 21] (Fig. 1a). Although ATF3 is a small protein containing only 181 residues, it harbors 17 lysine residues clustered proximal to where MDM2 binds (the BR domain) (Fig. 1a), suggesting that MDM2 might mediate more efficient transfer of Ub to ATF3 than to p53. We therefore asked if ATF3 needs to bind to MDM2 for p53 stabilization. Indeed, an ATF3 mutant lacking the MDM2-binding region (NLS-ΔBR) failed to increase the p53 level (Fig. 1b, lane 3 vs. lane 2). Although we previously ascribed the failure of ΔZIP in increasing p53 level (Fig. 1b, lane 4) to its loss of p53-binding activity [18], the ZIP domain contains the majority of lysine residues (Fig. 1a) and thus might also be required for ubiquitination. To address the concern that the deletion of a large region like BR might cause a structural change leading to an artifact, we swapped the ATF3 BR with that of JDP2 (Fig. 1c) and generated chimeric proteins. JDP2 is the closest family member of ATF3, and its predicted structure is highly similar to that of ATF3 in the BR-ZIP region (Fig. 1d). However, unlike ATF3, JDP2 did not bind MDM2 (Fig. 1e, lane 6) (but still bound p53 (Fig. 1f, lane 3)), was not a MDM2 substrate (Fig. 1g, lane 4), and could not stabilize p53 (Fig. 1b, lane 5). Consistent with our previous results that MDM2 binds to the ATF3 BR domain [21], the chimeric JDP2(AB) protein gained an ability to bind MDM2 as demonstrated by GST-pulldown (Fig. 1h, lane 6) and co-immunoprecipitation (co-IP) assays (Fig. 1i, lane 3). MDM2 could ubiquitinate this chimeric protein as efficiently as it did to ATF3 (Fig. 1j, lane 4). Importantly, this chimeric protein, like ATF3, increased the p53 level (Fig. 1k). These results thus support that the ATF3 BR region is required for p53 activation.

### ATF3 binding to MDM2 is required for p53 stabilization

**Fig. 2:**
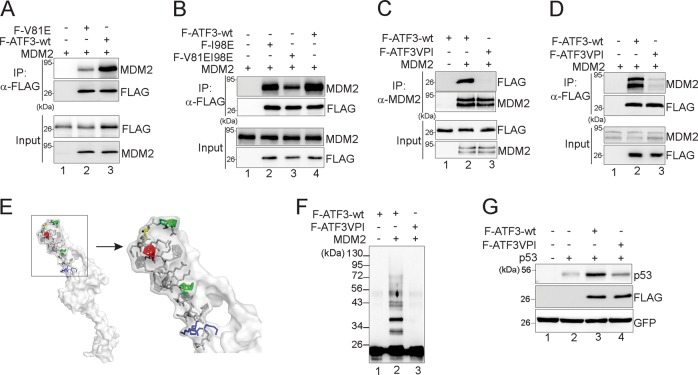
MDM2 binding is required for ATF3-mediated increase of p53 level. **a**, **b** FLAG-tagged V81E, I98E, or V81EI98E was coexpressed with MDM2 in H1299 cells, and subjected to FLAG-IP to determine MDM2-binding activity. H1299 cells transfected with FLAG-ATF3wt or FLAG-ATF3VPI (V81EP83DI98E) in the presence/absence of MDM2 were subjected to co-IP with the MDM2 antibody (**c**) or the anti-FLAG affinity gel (**d**) for western blotting as indicated. **e** A close look at the ATF3 BR structure and the ubiquitination sites. The three residues required for ubiquitination (K106, K107, and K108) are colored in blue. R88G is colored in red. **f** In vitro-translated FLAG-ATF3VPI and the wild-type control were subjected to in vitro ubiquitination assays. **g** H1299 cells were transfected as indicated for western blotting

As only a few residues are different between the sequence of JDP2 BR and that of ATF3 (Fig. 1c), we substituted the different residues (V81, P83, and I98) with corresponding JDP2 residues (Fig. 1c, labeled in red) in order to identify the residues required for MDM2 binding. Substituting V81 and/or I98 caused a significant decrease in MDM2-binding activity, but the mutants still bound MDM2 (Fig. 2a, b). Conversely, a mutant harboring substitutions at all of the three residues (ATF3-VPI) almost completely lost the MDM2-binding activity as shown by reciprocal co-IP assays (Fig. 2c, d, lane 3), suggesting that these three residues are required for MDM2 binding. Intriguingly, different models predicted by I-TASSER or QUARK [28, 29] reveal a similar “folded-back” conformation in the BR domain (Fig. 2e). P83 (colored by yellow) appears to be crucial for this conformation, and JDP2 BR indeed lacks a proline residue in the corresponding position. This folded-back configuration may ensure efficient binding of MDM2 to the two hydrophobic residues V81 and I89 (Fig. 2e, colored by green). As expected, MDM2 was unable to catalyze ubiquitination of ATF3-VPI (Fig. 2f, lane 3 vs. lane 2). Moreover, this MDM2 binding-deficient ATF3 mutant almost completely lost the ability to increase the p53 expression level (Fig. 2g). Taken together, our results support the notion that ATF3 needs to bind the MDM2 RING domain to activate p53.

### MDM2 catalyzes ATF3 ubiquitination at residues proximal to the MDM2-binding sites

**Fig. 3:**
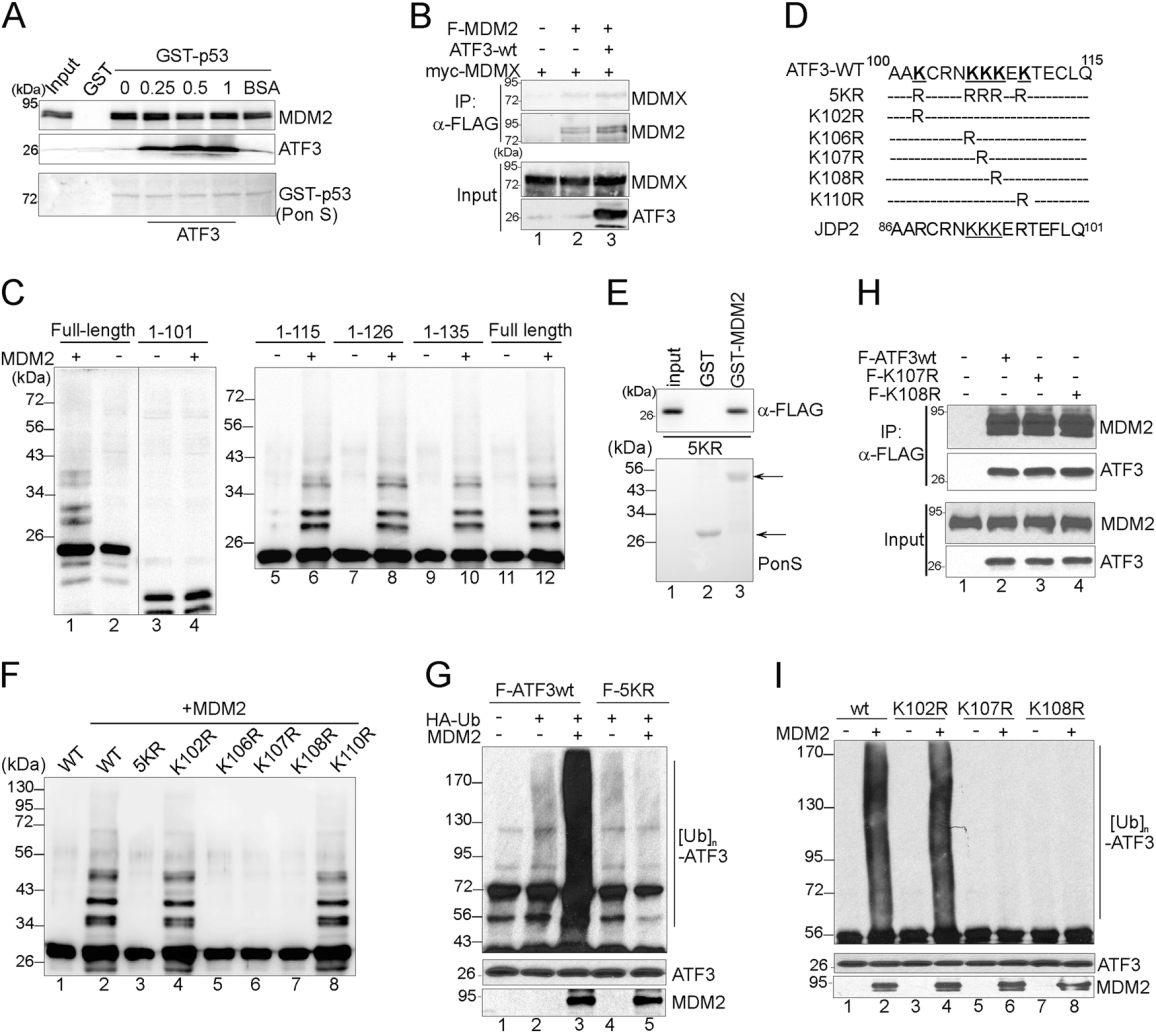
MDM2 catalyzes ATF3 ubiquitination at K106, K107, and K108. **a** In vitro translated MDM2 was incubated with immobilized GST-p53 in the presence/absence of increasing amounts of recombinant ATF3 for GST-pulldown assays. **b** H1299 cells were cotransfected with myc-MDMX, FLAG-MDM2, and/or ATF3-wildtype (ATF3-wt) for FLAG-IP assays. Bound proteins were subjected to western blotting to detect the MDM2–MDMX binding. **c** Indicated FLAG-tagged ATF3 C-terminal truncates were in vitro translated, and incubated with or without MDM2 for in vitro ubiquitination assays. Ubiquitinated proteins were detected with the FLAG antibody. **d** The sequence of ATF3 aa 100–115. The K to R substitutions used in this study are depicted. **e** FLAG-tagged 5KR was in vitro translated, and incubated with immobilized GST-MDM2 (384–491) for GST-pulldown assays. **f** Indicated FLAG-tagged ATF3 mutants were in vitro translated for in vitro ubiquitination assays. **g** FLAG-ATF3wt or 5KR was coexpressed with HA-ub and MDM2 in H1299 cells. Cell lysates were subjected to FLAG-IP followed by western blotting to detect ubiquitinated proteins with the HA antibody. **h** H1299 cells were transfected as indicated, and subjected to FLAG-IP to determine MDM2-binding activity. **i** FLAG-ATF3wt or mutants was coexpressed with HA-ub and MDM2 for in vivo ubiquitination assays

Although we have shown that the binding of ATF3 to MDM2 was required for ATF3-mediated p53 activation, this interaction did not result in dissociation of MDM2 from p53 (Fig. 3a) or MDMX (Fig. 3b). As ATF3 is likely a preferable substrate for MDM2, we next tested if ATF3 competitively inhibited MDM2-mediated p53 ubiquitination. This question can be answered by determining whether ATF3 mutants devoid of ubiquitination lose the ability to stabilize p53. Therefore, we generated several ATF3 C-terminal truncates and subjected them to in vitro ubiquitination assays [21] to identify the residues required for MDM2-mediated ubiquitination (Fig. 3c). These truncates contain the BR domain and thus were expected to bind MDM2. While deletion of the C-terminal 66 residues (aa 115–181) did not alter the ATF3 ubiquitination level (Fig. 3c, lanes 6, 8, and 10), further removing aa 102–115 completely abolished MDM2-mediated ubiquitination (Fig. 3c, lane 4), indicating that MDM2 likely ubiquitinated ATF3 at lysine residues residing in this region. Indeed, substituting the five lysine residues in this region to arginine (5KR) (Fig. 3d) did not block MDM2 binding (Fig. 3e, lane 3), but completely prevented ATF3 from ubiquitination catalyzed by MDM2 both in vitro (Fig. 3f, lane 3) and in vivo (Fig. 3g, lane 5). We also substituted these lysine residues with arginine individually (Fig. 3d) for in vitro ubiquitination assays. While MDM2 catalyzed efficient ubiquitination of K102R and K110R, substituting K107 or K108 with arginine, which did not alter the MDM2-binding activity (Fig. 3h), significantly impaired MDM2-mediated ubiquitination (Fig. 3f, lanes 6 and 7). K106R was also devoid of ubiquitination (Fig. 3f, lane 5), but a low ubiquitination level was detected with this mutant in a separate experiment. We confirmed that K107R and K108R were devoid of MDM2-mediated ubiuiqtination in vivo (Fig. 3i, lanes 6 and 8). These results thus indicated that MDM2 likely catalyzes the addition of ubiquitin chains to ATF3 at K106, K107, and K108. The observation that substituting one residue (i.e., K107 or K108) was sufficient to cause a complete obliteration of MDM2-mediated ubiquitination is not without precedent [30], suggesting that the transfer of ubiquitin to one residue likely primes ubiquitination of the other. While a consensus “ubiquitin motif” has not been able to be identified, it is noteworthy that K106, K107, and K108 locate proximal to the residues where MDM2 binds to (Fig. 2e, colored by blue). However, proximity in sequence does not warrant ubiquitin transfer as K102 did not appear to be required for ubiquitination. It is also worth noting that JDP2 has three lysine residues (K92, K93, and K94) located at positions corresponding to that of K106, K107, and K108 in ATF3 (Fig. 3d), which provides the sites necessary for ubiquitination when JDP2 was engineered to bind MDM2 through replacing its BR with that of ATF3.

### ATF3 mutants devoid of ubiquitination fail to block p53 ubiquitination and increase p53 level

**Fig. 4:**
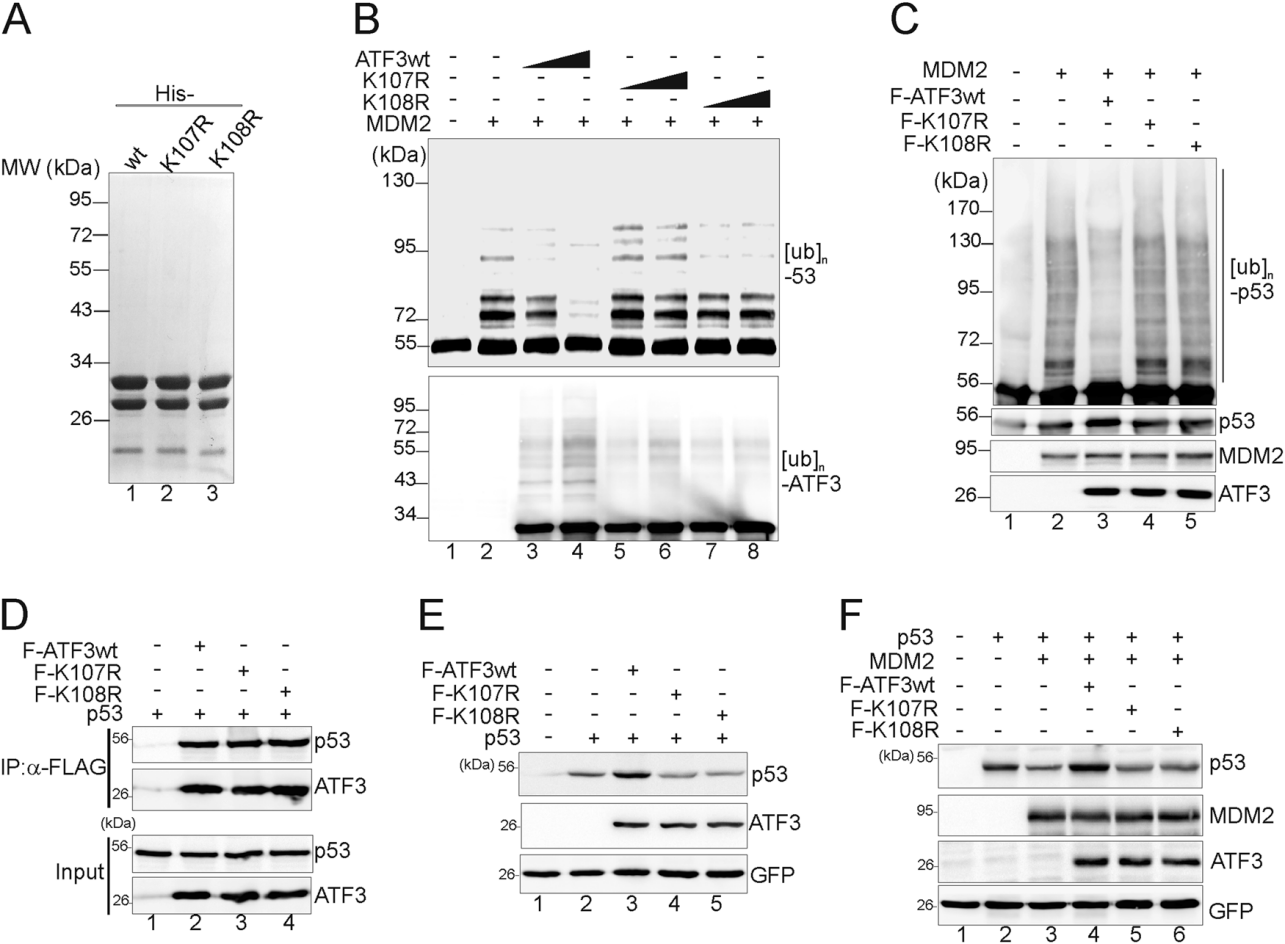
ATF3 mutants devoid of ubiquitination fail to increase the p53 expression level. **a** Histidine-tagged ATF3wt, K107R, and K108R were purified with Ni^+^-NTA agarose, and their purity was determined by SDS-PAGE. **b** In vitro translated p53 was incubated with recombinant ATF3wt, K107R, or K108R for in vitro ubiquitination assays. After determining the p53 ubiquitination level using the p53 antibody, the same blot was stripped and incubated with the ATF3 antibody to determine the ATF3 ubiquitination level. **c** H1299 cells were transfected with FLAG-tagged ATF3wt, ATF3 mutants, HA-ub, and MDM2 as indicated for IP with the HA antibody. The p53 ubiquitination level was determined with the p53 antibody. **d** Transfected H1299 cells were subjected to FLAG-IP followed by western blotting to determine the p53-binding activity. **e** H1299 cells were transfected as indicated for western blotting. **f** H1299 cells were transfected with p53 and MDM2 in the presence/absence of ATF3wt or mutants for western blotting

The observations that ATF3 binds to MDM2 RING and was ubiquitinated at residues proximal to where MDM2 binds suggest that MDM2-mediated ATF3 ubiquitination could be more efficient and thus may competitively inhibit p53 ubiquitination. To test this, we purified recombinant wild-type (ATF3wt) and ubiquitination-devoid K107R and K108R (Fig. 4a) for in vitro p53 ubiquitination assays. Consistent with our previous results [18], ATF3wt dramatically blocked p53 ubiquitination catalyzed by MDM2 (Fig. 4b, lanes 3–4 vs. lane 2). Conversely, despite that they retained the p53-binding (Fig. 4d) and the MDM2-binding activity (Fig. 3h), K107R and K108R failed to prevent p53 from MDM2-mediated ubiquitination in the in vitro ubiquitination assays (Fig. 4b, lanes 5–8). The ability of these mutants to block p53 ubiquitination in vivo was lost as well (Fig. 4c, lanes 4–5 vs. lane 3). It is noteworthy that ATF3wt was ubiquitinated by MDM2 while it blocked p53 ubiquitination (Fig. 4b, lanes 3–4), in line with the notion that ATF3 competitively inhibited p53 ubiquitination. As the p53 level is mainly regulated by its ubiquitination level [5, 31], it was not surprising that K107R and K108R were unable to increase the level of coexpressed p53 (Fig. 4e, lanes 4–5 vs. lane 3). Similarly, while ATF3wt prevented p53 from MDM2-mediated degradation, the ubiquitination-devoid ATF3 mutants lost this ability (Fig. 4f, lanes 5–6 vs. lane 4). Therefore, ATF3-mediated prevention of p53 ubiquitination and degradation was likely a consequence of competitive ubiquitination between ATF3 and p53.

### ATF3 mutants devoid of ubiquitination have a defect in activating p53 in response to DNA damage

**Fig. 5:**
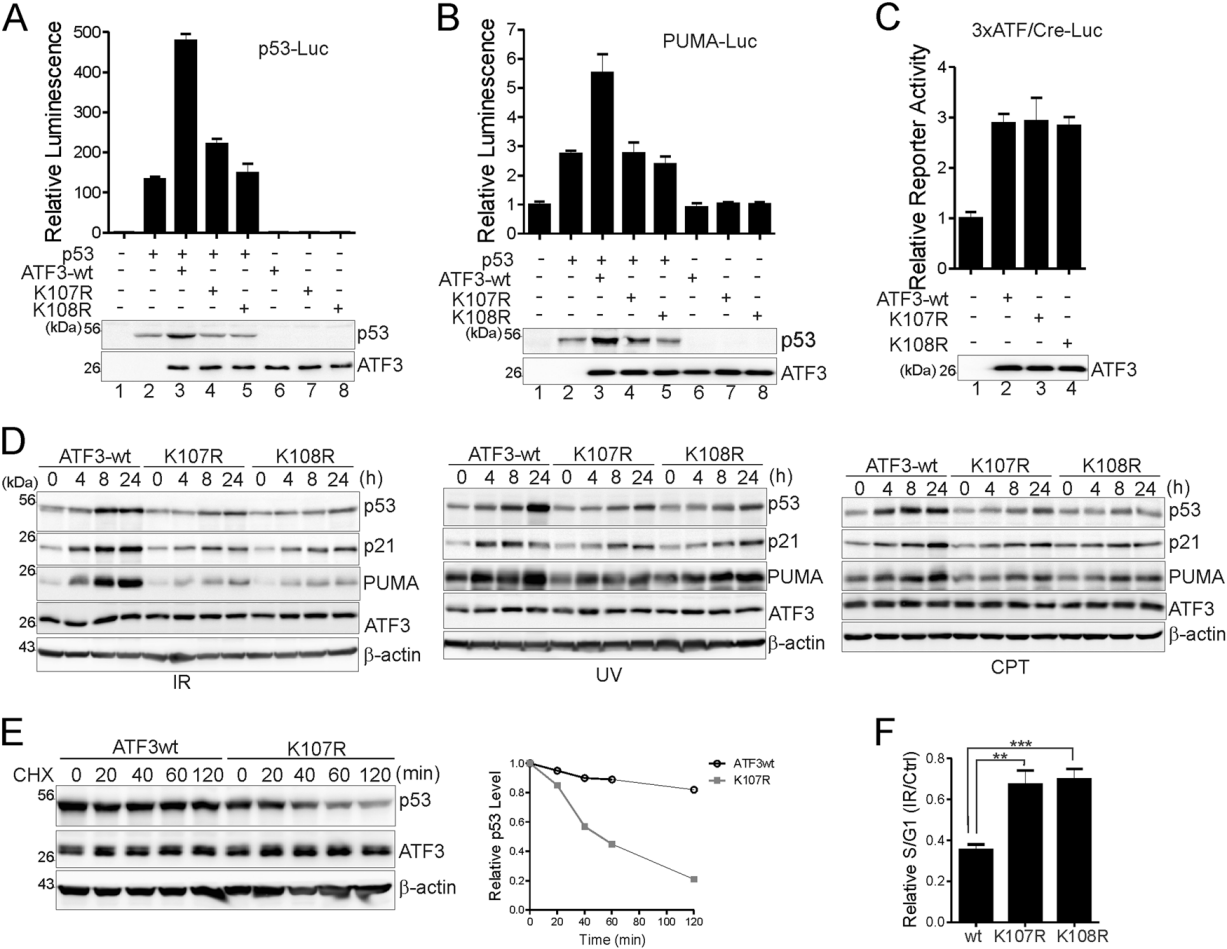
ATF3 ubiquitination is required for p53 activation. **a**, **b** H1299 cells transfected with p53-Luc or PUMA-luc, pRL-TK, p53, ATF3, and ATF3 mutants as indicated were subjected to dual luciferase activity assays. **c** H1299 cells were transfected with 3×ATF/Cre-Luc, pRL-TK and ATF3wt, or mutants for dual luciferase activity assays. **d** Indicated reconstituted U2OS cells were treated with IR (10 Gy), UV (10 J/m^2^), or CPT (1.5 μM) for different time and lysed for western blotting. **e** ATF3wt and K107R cells were exposed to UV (10 J/m^2^) for 2 h, and then treated with 100 μg/ml of cycloheximide (CHX) for different time for western blotting. The p53 level was quantitated by densitometrical analysis, normalized to the β-actin level, and plotted. **f** Indicated cells were treated with 10 Gy of IR for 24 h, and stained with PI for cell cycle analysis. The data were presented as mean ± SD. **p < 0.01; ***p < 0.001; Student’s *t* test (two-sided)

To understand the functional significance of our findings, we determined whether the ubiquitination-devoid K107R and K108R mutants conferred impaired p53 activation. Consistent with its ability to block p53 ubiquitination, ATF3wt promoted p53 to transactivate a synthetic p53-activity promoter (p53-Luc) (Fig. 5a, lane 3), or a PUMA promoter (Fig. 5b, lane 3). Conversely, neither K107R nor K108R promoted p53-mediated transactivation of these promoters (Fig. 5, b, lanes 4–5), likely due to impaired elevation of p53 level (see immunoblots in Fig. 5a, b). Note that substituting these lysine residues did not alter ATF3 transcriptional activity (Fig. 5c) nor its nuclear localization (Supplementary Fig. S1A). The result that ATF3wt transactivated rather than repressed the synthetic ATF/Cre reporter (3×ATF/Cre-Luc) was somewhat unexpected [32], but ATF3 binding to DNA can result in either activation or repression of transcription in a context-dependent manner [13]. To determine effects of the ATF3 mutants on p53 activation in vivo, we knocked out the endogenous *ATF3* gene by CRISPR/Cas9 from U2OS cells [27] and reconstituted the cells with ATF3wt, K107R, or K108R. The derived cell lines expressed ATF3wt, K107R, or K108R at a comparable level (Fig. 5d). The ATF3wt cells retained an active p53 response to DNA damage, evidenced by elevated expression of p53 and its target genes (e.g., p21 and PUMA) upon treatments with DNA-damaging agents including IR, ultraviolet light (UV), or camptothecin (CPT) (Fig. 5d). Conversely, the increase of p53 activity induced by these agents was dramatically impaired in the K107R- and K108-reconsituted cells (Fig. 5d). While ATF3wt increased the p53 half-life in response to DNA damage, we confirmed that K107R largely lost this activity (Fig. 5e), thereby demonstrating that ATF3 ubiquitination is required for p53 stabilization. Of note, although MDM2-mediated degradation of MDMX is important for p53 activation upon DNA damage [33–35], ATF3 ubiquitination did not appear to affect this event as DNA-damaging agents induced MDMX degradation in the K107R cells to the same extent as they did in the ATF3wt cells (Supplementary Fig. S1B). We also subjected γ-irradiated cells to cell cycle analysis to determine whether loss of ubiquitination impaired p53 function. IR mainly caused a G2 arrest in U2OS cells, which is p53-independent [36, 37]. However, KO of p53 by CRISPR/Cas9 (sgp53) induced a significant increase of S phase cells and a concomitant decrease of G1 phase cells as compared with wild-type cells post IR (Supplementary Fig. S2), in line with the notion that p53 functions to induce G1 arrest after cells escape from the earlier G2 arrest [36, 37]. Consistent with the impaired p53 activation (Fig. 5d), more K107R/108R-reconstituted cells stayed in S phase as compared with ATF3wt cells 24 h after IR (Fig. 5f), indicating that the p53 function was indeed impaired in those cells expressing ubiquitination-devoid ATF3 mutants. These results thus support that MDM2-mediated ubiquitination of ATF3 was required for p53 activation in the DNA damage response.

### A cancer-derived ATF3 mutant lacks ubiquitination and is defective in p53 activation

**Fig. 6:**
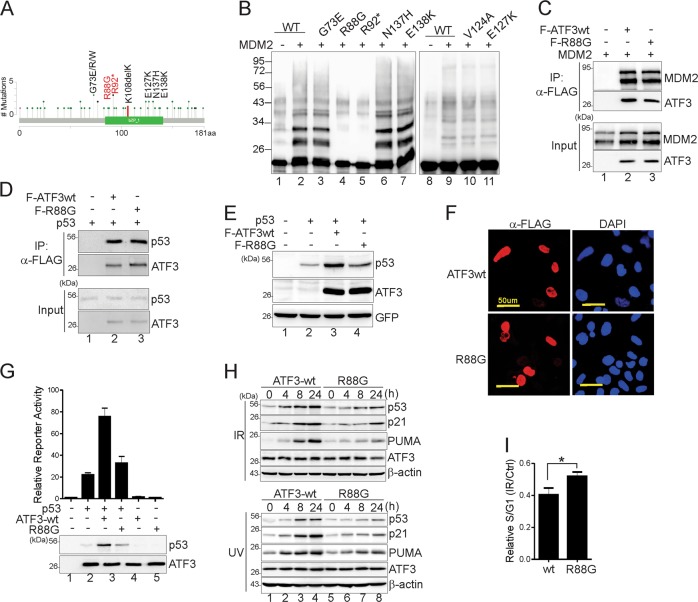
A cancer-derived ATF3 mutant is unable to activate p53 in the DNA damage response. **a** A cBioPortal view of *ATF3* mutants found in human cancers. The K108delK mutant was identified by COSMIC. **b** Indicated ATF3 mutants were in vitro translated, and subjected to in vitro ubiquitination assays. The ubiquitination level was determined by the FLAG antibody. H1299 cells were transfected with FLAG-ATF3wt, FLAG-R88G, MDM2, or p53 as indicated, and subjected to FLAG-IP assays to determine the MDM2-binding (**c**) and the p53-binding activity (**d**). **e** H1299 cells transfected with p53 and/or FLAG-ATF3wt or FLAG-R88G were subjected to western blotting. **f** ATF3-knockout U2OS cells were transfected with FLAG-ATF3wt and FLAG-R88G, and subjected to cytoimmunostaining with the FLAG antibody. **g** H1299 cells transfected with p53-Luc, pRL-TK, p53, ATF3wt, or R88G as indicated were subjected to dual luciferase activity assays. **h** ATF3wt and R88G-reconsituted U2OS cells were treated with 10 Gy of IR or 20 J/m^2^ of UV for western blotting. **i** Indicated reconstituted U2OS cells were exposed to 10 Gy of IR for 24 h, followed by cell cycle analysis using flow cytometry. The data were presented as mean ± SD. *p < 0.05, Student’s *t* test (two-sided)

Although ATF3 plays a context-dependent role in cancer progression and metastasis [14, 15], *Atf3*^*−/−*^ mice are tumor prone in part due to impaired p53 activation [16]. To provide genetic evidence supporting the importance of ATF3 ubiquitination to p53 activation, we searched public cancer mutation databases (cBioPortal and COSMIC) for *ATF3* mutations in human cancers. In spite of a low *ATF3* mutation rate (0.1%), a colon cancer was found to harbor an *ATF3* mutation (K108delK) lacking K108—a residue required for ubiquitination (Fig. 6a). Moreover, a pancreatic cancer and two uterine cancers harbor a mutation (R92*) that generates a stop codon after R92 and thus lacks all of the ubiquitination sites (Fig. 6a). Intriguingly, all of these cancers carry a wild-type p53 gene, suggesting that *ATF3* mutations might contribute to impaired p53 activity in these cancers. To further address the effects of ubiquitination-devoid *ATF3* mutations on p53 activity in cancer, we subjected several missense mutations in the region flanking the MDM2-binding sites or the ubiquitination sites for in vitro ubiquitination assays. While we confirmed that R92* was devoid of ubiquitination (Fig. 6b, lane 5), MDM2 did not ubiquitinate R88G (lane 4)—a mutation found in a papillary renal carcinoma with wild-type *TP53*. As the R88G mutant retained its MDM2-binding activity (Fig. 6c), it was unlikely that impaired ubiquitination was a consequence of dissociation from MDM2. Probably, the positive-charged lysine residue (Fig. 2e, red) might be required for contacting with E2-Ub for Ub transfer. Although the mutation did not affect the ATF3–p53 interaction (Fig. 6d), nor its nuclear localization (Fig. 6f), R88G failed to increase the p53 level (Fig. 6e, lane 4 vs. lane 3), nor promote p53 to transactive its responsive promoter (Fig. 6g, lane 4 vs. lane 3), indicating that this cancer-derived, ubiquitination-devoid mutant lost the ability to activate p53. Indeed, substituting ATF3wt with R88G in U2OS cells dramatically impaired the increase of p53, p21, and PUMA expression level induced by IR and UV (Fig. 6h). As a consequence, IR-induced, p53-dependent G1 arrest was also significantly impaired in R88G-reconstituted cells. These results have demonstrated that a cancer-derived ATF3 mutant devoid of ubiquitination but retaining the p53- and the MDM2-binding activity could cause defective p53 activation upon DNA damage. Therefore, while epigenetic silencing may cause frequent downregulation of *ATF3* expression in cancer [15, 16, 38], mutations at this putative tumor suppressor gene can also impair its ability to activate p53 thereby leading to tumorigenesis.

## Discussion

Loss of p53 activation upon genotoxic stress can lead to tumorigenesis. While blocking MDM2-mediated ubiquitination is central to p53 activation, a number of mechanisms that can restrict MDM2 from accessing p53 have been discovered [3]. However, little is known about the mechanism(s) by which the MDM2 catalytic activity is regulated. In this study, we have shown that MDM2-mediated ubiquitination of ATF3 is required for p53 activation. Our results thus support a model, in which ATF3 competitively inhibits MDM2-mediated ubiquitination of p53 thereby activating the tumor suppressor. Although ATF3 is a substrate of MDM2 [21], DNA damage rapidly increases the cellular ATF3 level mainly through inducing *ATF3* transcription [39, 40]. Thus, transcriptionally-induced ATF3 could serve as an “ubiquitin trap” to activate p53 in response to genotoxic challenges. To our knowledge, this is the first report revealing that p53 can be activated by competitive ubiquitination. As an array of proteins can interact with MDM2 and are ubiquitinated by MDM2 [41], competitive ubiquitination could be used as a common mechanism for p53 activation upon genotoxic stress. Ribosomal protein S7, for instance, is a MDM2 substrate that can inhibit p53 ubiquitination and stabilize the latter [42]. While ATF3 bound to MDM2 at the catalytic RING domain, it was ubiquitinated at sites located close to where MDM2 binds (Fig. 2e). Although it remains elusive whether such spatial proximity is required for ATF3-mediated competitive inhibition of p53 ubiquitination, other MDM2-binding partners (e.g., HIPK2) can bind to the MDM2 RING domain as well [41]. However, sites at which these MDM2-binding proteins are ubiquitinated often remain undefined. Interestingly, although MDM2 can also catalyze MDMX ubiquitination [33, 34], ATF3 ubiquitination did not appear to block MDMX ubiquitination as DNA damage-induced MDMX degradation was not affected by K107R (Supplementary Fig. S1B). Given that MDMX also binds MDM2 at its RING domain, this result lends a support to our hypothesis that ATF3-mediated competitive inhibition of p53 ubiquitination is due to the spatial proximity of ATF3 to E2s. It is important to note that, our findings that ATF3 was ubiquitinated at sites proximal to where MDM2 binds suggest that it has finally become possible to crystalize the MDM2 RING domain in a complex with its substrate, thereby allowing for structural dissection of the ubiquitination reaction catalyzed by MDM2. Given that MDM2 is the major E3 ubiquitin ligase that dictates the p53 level in cells, a better understanding of how MDM2 catalyzes the transfer of ubiquitin would provide the ground for the development of novel strategies for therapeutic activation of p53 in cancer.

Among a range of strategies that have been developed for therapeutically activating p53 in cancer [43], small molecules (e.g., nutlin derivatives) that can bind MDM2 and block its binding to p53 have proven to be effective in elevating the cellular p53 level and inducing apoptosis in various cancer types in clinical trials. However, these p53-activating agents often exhibit dose-limiting adverse effects, which are most-commonly related to gastrointestinal and hematological disorders, and could be caused, at least in part, by elevated MDM2 expression subsequent to p53 activation induced by the small molecules. Indeed, while MDM2 is a well-characterized p53 target gene, MDM2 has many p53-independent activities that are associated with its E3 ubiquitin ligase activity [22, 44]. Our results that ATF3 could competitively inhibit the catalytic activity of MDM2 suggest that ATF3 could also inhibit ubiquitination of other MDM2 substrates and thus alleviate undesired effects caused by p53 activation. Therefore, harnessing this newly-discovered mechanism would lead to a new anticancer strategy that may activate p53 without the expense of undesired effects caused by elevated MDM2 expression [45]. Interestingly, as a common stress sensor, ATF3 expression can be induced by a large number of therapeutic agents (e.g., camptothecin and cisplatin). However, ATF3 induction by these agents are often transient, likely due to the fact that ATF3 can bind to its own promoter and repress its own expression [46]. There is thus a need of identifying small molecules that can sustainably increase the ATF3 level in cancer cells. Although ATF3 was reported to decrease the p53 mRNA level in human umbilical vein endothelial cells and human keratinocytes [47, 48], it is worth noting that this contrary effect likely limits to specific cell types as we have never seen such an effect in either primary or cancer cells that we intensively investigated. Moreover, the fact that the p53 activity is mainly regulated at the posttranscriptional level [49] suggests that ATF3-mediated stabilization of p53 could override any possible adverse effect on p53 transcription.

Previously, we ascribed the effect of ATF3 on p53 ubiquitination to its binding to the p53 C-terminus based on the results that the ATF3 ZIP-deleting mutant (ΔZIP) failed to stabilize p53 [18]. However, the ZIP domain (aa 102–139) not only mediates p53 binding [18], but also contains the lysine residues (K106, K107, and K108) responsible for ubiquitination. As ΔZIP lacks ubiquitination as well, it becomes clearer now that it is more likely that ATF3 stabilizes p53 through binding to MDM2 and competitively inhibiting its catalytic activity. Indeed, while ATF3 binding to MDM2 could increase the p53 stability (Figs. 1, 2), the ubiquitination-devoid ATF3 mutants (e.g., K107R, K108R, and R88G) unable to activate p53 retained the binding affinity to p53 (Figs. 4d, 6d). Interestingly, JDP2, the closest family member of ATF3 that weakly binds p53, failed to stabilize p53 as well. As the BR-ZIP domain responsible for DNA binding is highly similar between ATF3 and JDP2, it is often assumed that JDP2 has similar functions as ATF3 and can compensate for ATF3 loss in cells [50]. However, our results indicate that JDP2 differs largely from ATF3 in its ability to bind MDM2 and activate p53. Therefore, these two related proteins may play different roles in tumorigenesis and other physiological/pathological events.

## Supplementary information

Supplementary Figure legends

Figure S1

Figure S2
