# Supplementary material for: Competitive ubiquitination activates the tumor suppressor p53
Source: Cell Death Differ. 2019 Dec 2;27(6):1807–18. doi: 10.1038/s41418-019-0463-x (PMC7244561; doi:10.1038/s41418-019-0463-x)
Supplement: Supplementary file 1 — Supplementary Figure legends [file 41418_2019_463_MOESM1_ESM.docx]

Supplementary Figure legends

Fig S1. **ATF3 mutants retains its nuclear localization and does not affect MDMX degradation induced by DNA damage.** (**A**) ATF3-knockout U2OS cells were transfected with FLAG-tagged ATF3wt, K107R, or K108R for cytoimmunostaining with the FLAG antibody. (**B**) Cells reconstituted with ATF3-wt or K107R were treated with 10 Gy of IR or 1.5 μM of CPT for Western blotting as indicated.

Fig S2. **Knockout of p53 impairs IR-induced G1 arrest in U2OS cells.** (**A**) p53 expression was knocked out in U2OS cells by CRISPR/Cas9. The cells were treated with 10 Gy or IR for 24 h for Western blotting. (**B**) U2OS-wt and p53-knockout cells were exposed to 10Gy of IR. 24 h later, the cells were subjected to flow cytometry analysis.
