# Supplementary figures and images for: Competitive ubiquitination activates the tumor suppressor p53
Source: Cell Death Differ. 2019 Dec 2;27(6):1807–18. doi: 10.1038/s41418-019-0463-x (PMC7244561; doi:10.1038/s41418-019-0463-x)

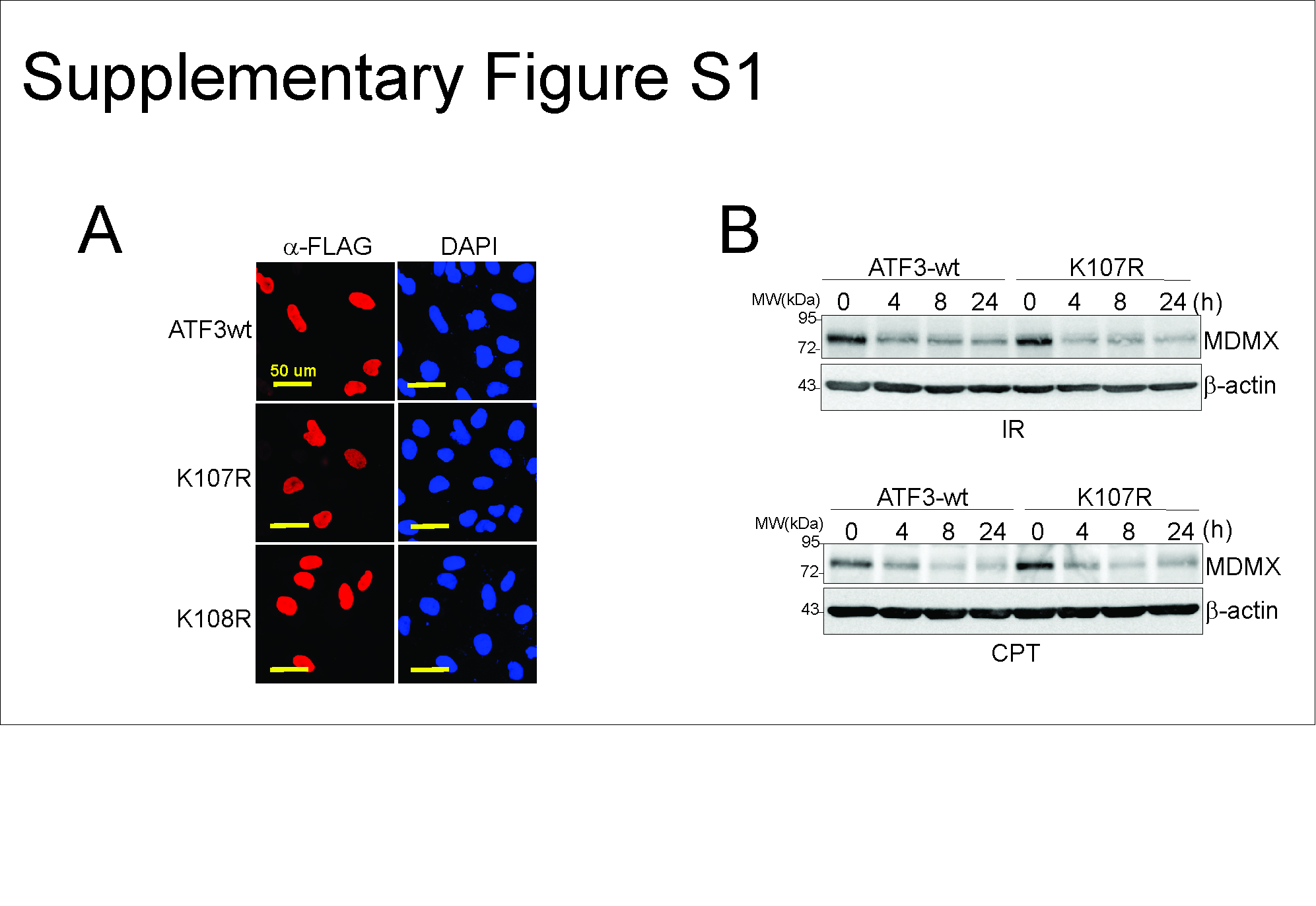

Supplement: Supplementary file 2 — Figure S1 [file 41418_2019_463_MOESM2_ESM.tif]

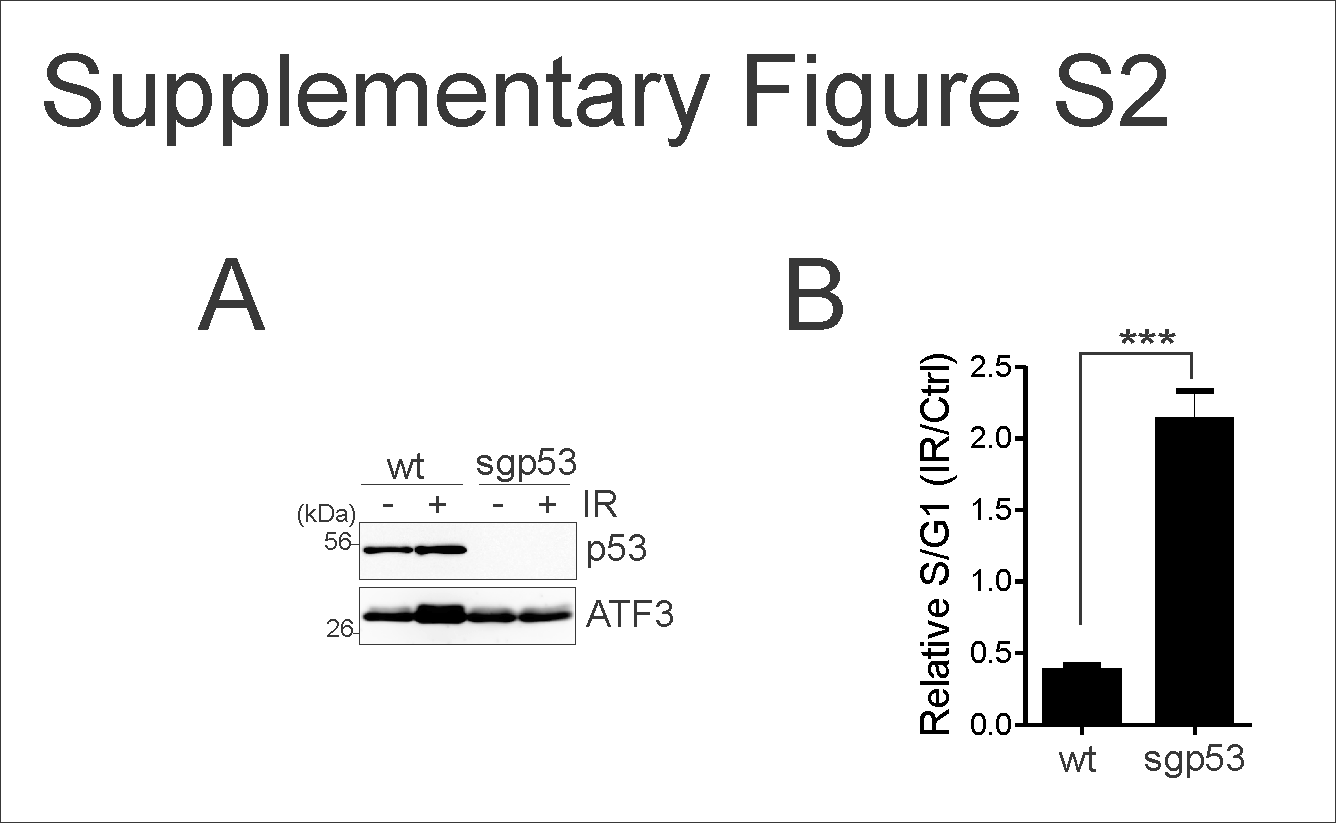

Supplement: Supplementary file 3 — Figure S2 [file 41418_2019_463_MOESM3_ESM.tif]
